# Sentinel Lymph Node Mapping in Endometrial Cancer: A Comprehensive Review

**DOI:** 10.3389/fonc.2021.701758

**Published:** 2021-06-29

**Authors:** Lirong Zhai, Xiwen Zhang, Manhua Cui, Jianliu Wang

**Affiliations:** ^1^ Department of Gynecology and Obstetrics, Peking University People’s Hospital, Beijing, China; ^2^ Department of Gynecology and Obstetrics, The Second Hospital of Jilin University, Changchun, China

**Keywords:** sentinel lymph node, endometrial cancer, lymphadenectomy, low-volume metastases, high risk, sentinel lymph node biopsy, sentinel lymph node mapping

## Abstract

Endometrial cancer (EC) is known as a common gynecological malignancy. The incidence rate is on the increase annually. Lymph node status plays a crucial role in evaluating the prognosis and selecting adjuvant therapy. Currently, the patients with high-risk (not comply with any of the following: (1) well-differentiated or moderately differentiated, pathological grade G1 or G2; (2) myometrial invasion< 1/2; (3) tumor diameter < 2 cm are commonly recommended for a systematic lymphadenectomy (LAD). However, conventional LAD shows high complication incidence and uncertain survival benefits. Sentinel lymph node (SLN) refers to the first lymph node that is passed by the lymphatic metastasis of the primary malignant tumor through the regional lymphatic drainage pathway and can indicate the involvement of lymph nodes across the drainage area. Mounting evidence has demonstrated a high detection rate (DR), sensitivity, and negative predictive value (NPV) in patients with early-stage lower risk EC using sentinel lymph node mapping (SLNM) with pathologic ultra-staging. Meanwhile, SLNM did not compromise the patient’s progression-free survival (PFS) and overall survival (OS) with low operative complications. However, the application of SLNM in early-stage high-risk EC patients remains controversial. As revealed by the recent studies, SLNM may also be feasible, effective, and safe in high-risk patients. This review aims at making a systematic description of the progress made in the application of SLNM in the treatment of EC and the relevant controversies, including the application of SLNM in high-risk patients.

## Introduction

Endometrial cancer (EC) is known as a common female genital malignancy with rapidly increasing incidence these years. In 2021, there will be an estimated 66,570 new cases and 12,940 deaths, making uterine cancer the second most prevalent cancer in women in U.S. after breast cancer (1). Surgery is the mainstay for treatment include total hysterectomy + bilateral salpingooophorectomy + pelvic lymphadenectomy +/− para-aortic lymphadenectomy (TH+BSO+PLAD+/−PALAD). LAD represents a significant component of comprehensive staging for patients with EC. However, studies have revealed that LAD may not be conducive to the prognosis of EC patients (2, 3). Besides, the selective lymphadenectomy (SLAD) based on “Mayo criteria” shows a high sensitivity with a low specificity (4), and 80% of the high-risk patients undergo excessive lymph node dissection (5). Additionally, lymph node resection brings a series of complications like vascular nerve injury, lymphedema, lymphatic cysts, and so on (6). Therefore, SLNM or sentinel lymph node biopsy (SLNB) can be effective in addressing this drawback. SLNM does not compromise patient outcome by providing enough information on lymph node directing adjuvant therapy (7), meanwhile, it improves the quality of life by shortening operation time and reducing complications (8). This review is to give a comprehensive view of the application of SLNM in EC, thus providing further choices regarding the lymph node dissection.

## Disputes About LAD for EC

The I–IV staging system of EC was initiated in 1962 and transferred from clinical staging to surgical pathologic staging in 1988 (9). Furthermore, staging protocol was re-edited in 2009 for setting IIIC1 as positive pelvic lymph nodes, while IIIC2 refers to the positive para-aortic lymph nodes (10). LAD is an essential part of staging surgery for it provides the lymph node information thus indicating adjuvant therapy, evaluating prognosis, and acting as a therapeutic role. Patients with pelvic or para-aortic lymph node metastasis has dramatically decreased survival rate (10). Additionally, it is believed that LAD eliminates not only existing metastases but also occult or potential metastasis (11). Large retrospective studies showed that LAD is associated with prolonged survival outcome, especially in high-risk EC (12).

However, the therapeutic role and survival benefit of LAD have been in controversial in recent years with the publication of a series of high-quality research. Two large randomized controlled clinical trials (RCT) in 2008 and 2009 included 514 and 1408 patients with EC found no statistical significance in PFS and OS between LAD or not (13, 14). Though the two studies are blamed for varying design defects, such as LAD group, did not perform PALAD, the two groups of high-risk patients were not balanced, the proportion of low-risk patients was larger, and adjuvant therapy was not standardized, but it did arouse intensive debates (15). A more recent multicenter study performed by Bougherara et al. (3) demonstrated that LAD brings no survival benefits in intermediate-risk EC group and Zhang et al. (16) analyzed SEER databases and found that after balancing mixing factors, LAD has no survival difference for patients in clinical stage IA with any histologic grade. Besides, LAD increased the incidence of intraoperative complications (prolonged operation time, excessive bleeding, vascular nerve injury, etc.) and post-operative complications (lymphedema, lymphocyst, intestinal obstruction, deep venous thrombosis), thus affecting the quality of life for patients (17). Beesley et al. (18) followed up 643 EC patients and found that the incidence of lymphedema was related to the number of lymph nodes removed, the risk climbed to 50% when cutting more than 15 lymph nodes. Volpi et al. pointed out that LAD and PALAD are independent risk for lymphedema and lymphocele (6).

At present, the most commonly used strategy is “SLAD” according to “Mayo Criteria” proposed by Mariani et al. (19) in 2000. That is to say, LAD could be omitted in low-risk group (meet all of the following conditions: (1) endometroid type, grade G1 or G2; (2) myometrial invasion< 1/2; and (3) tumor diameter < 2 cm), while LAD should be applied in high-risk group (not in accordance with any of the above). However, evidence has confirmed the ability of the method to identify patients with low risk (1%–2.4%) or high risk [11.4%–19% (5, 20, 21)] with a high sensitivity of 90%, which remains the most sensitive method in determining which patients can be omitted from LAD (22), while the specificity is only 36% (4). Nearly 80% of the high-risk group without metastases undergo LAD. In addition, the criteria depend on intraoperative frozen section (FS) and the coincidence rate with postoperative pathology declines when the histology grade and myometrial invasion degree increases, which result in approximately 18% of EC patients up-staged when final pathologic reports come (23, 24).

Therefore, the emergence of SLNM provides an alternative for both systemic LAD and SLAD. Not only does it reduce complications and improve the quality of life of patients, it provides sufficient staging information for evaluating prognosis and guiding adjuvant therapy. Most importantly, it seems not to compromise the survival outcomes of EC patients.

## The Concept and Origin of SLN

SLN refers to one or several lymph nodes that first receive lymphatic fluid from an organ or regional tissue, or the first lymph node that is impacted by the lymphatic metastasis of the primary malignant tumor through the regional lymphatic drainage pathway, thus indicating the involvement of the whole drainage area (25). Theoretically, if SLN is negative, lymphatic metastasis of the drainage area does not occur yet, thus avoiding LAD with following surgical trauma (11). If SLN is found positive in FS, the LAD can be performed directly during the operation. If the H&E staining and/or ultra-staging of SLN is positive after surgery, patients can either choose adjuvant therapy or a second operation. It is noted that FS of SLN is not a routine in many institutions due to its cost and inaccuracy in finding low volume metastatic disease (LVMD) (10, 26), while some send corpus uterine for FS assessment when SLN map failure occurs (27), which is also mentioned in the latest NCCN guideline.

In 1960, Gould et al. first discovered and defined SLN in parotid carcinoma (28). In 1977, Cabanas first used SLN lymphangiography in penile cancer (29). SLNM gradually became a routine procedure for the treatment of breast cancer and skin melanoma. Burke was the first to perform SLNM on 15 patients with EC back in 1996 (30). In the most recent decade, SLN has developed rapidly in EC and has been applied to the treatment of gynecological tumors such as vulvar cancer, cervical cancer, and EC. By resecting two to four high-quality lymph nodes, SLNM may have the same diagnostic advantages as LAD and minimize surgical injuries.

## The Technique Advances of SLNM

### Detection Method

At the present time, SLN detection methods include blue dye method, radionuclide tracing method, indocyanine green (ICG), carbon nanoparticle (CNP), and combination method.

Blue dye method, also known as bioactive dye tracing method, including methylene blue, isosulfur blue, and patent blue. The dye can reach lymphatic vessels and lymph nodes around the tumor, and SLN is the first lymph node to show color. The method features simplicity and cost-effectiveness. However, the blue dye can diffuse to parametrial area thus interfering with the discovery of regional SLN (31). Some methylene blue may leak into the capillaries, resulting in reduced dye volume in lymphatic pathway and decreased SLN DR (32). Also, the risk of allergy cannot be ignored (33).

Radioactive tracers like technetium(Tc)-99^m^ can remain highly concentrated in the SLN, and emit gamma-rays, which will be detected by gamma detector and single-photon emission computed tomography (SPECT-CT). Radioisotopes can transmit signals through deep tissues. However, the higher cost of detection and imaging equipment, inconvenience, and potential radioactive contamination limit its use (31). The cervical injection site can also stimulate gamma detectors, which makes it difficult to be distinguished from parametrial lymph nodes (34).

**Figure 1:**
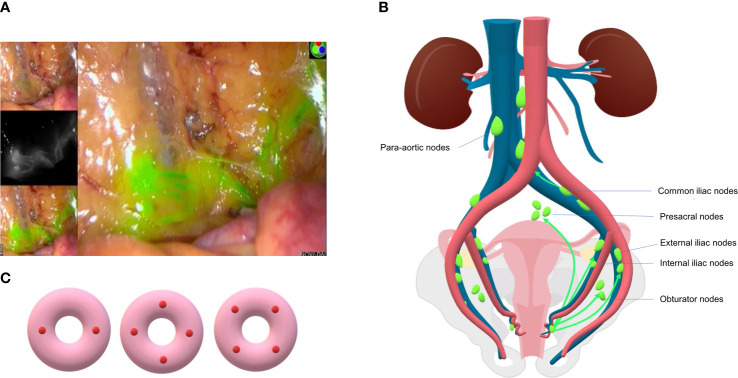
**(A)** SLN and lymphatic vessel mapped in surgery using ICG dye (Liaoning Pharmaceutical Co., Ltd.) and intraoperative fluorescence imaging system (PC9000, Novadaq Technologies Inc.). **(B)** Common lymphatic drainage pathway of endometrial cancer. SLNs are mostly located in external iliac and obturator region and less commonly in presacral and common iliac area. **(C)** Three patterns of cervical injection sites of SLNM: two sides or four quadrants.

ICG fluorescence labeling relies on ICG, a near-infrared fluorescent dye, to drain through lymph nodes and stimulate fluorescence under near-infrared light (700-900 nm) (11) (**Figure 1A**). It is the most recommended tracer in researches and guidelines, especially for patients with minimally invasive surgery and obesity, due to its highest DR and bilateral detection rate (BDR) (35–37). A randomized non-inferiority trial of 180 patients with uterine and cervical cancer showed that, ICG detected 97% of the total lymph nodes dissected whereas blue dye identified only 47% (38). Recent research from Germany compared ICG with blue dye in EC and cervical cancer, as a result, ICG improved the DR (78% *vs.* 61%, p=0.006) and therefore decreased the LAD rate from 28% to 9% (p=0.001) when mapping failure occurs (39). However, the method relies on near-infrared device (40). Also, ICG enhanced the visualization of lymphatic channels, which leads to an increase in “empty node,” which may be compromised by the combination of ICG and Tc-99m (41). Though the adverse reaction rate is extremely low (0.07% to 0.5%) (42), it should be avoided in patients with iodine allergy and liver failure, since it is completely metabolized through liver (10).

CNP suspension derives from carbon nanoparticles with a diameter of 150 µm (43). It enters the lymphatic system by macrophage and is excreted through the respiratory and gastrointestinal tract (44). It owns the advantages of unique lymphatic system tendency, small-size, fast diffusion, and long-lasting color rendering (43). Meanwhile, it can adsorb anti-cancer drugs and is difficult to leak out when lymphatic channels are cutoff intraoperatively (43). There are no adverse reactions reported yet, and the DR is quite high. Data from our hospital showed that the combination of CNP and ICG resulted a higher BDR of SLN in cervical and endometrial cancer comparing to CNP or ICG alone (p<0.05) (45, 46).

The combined method is usually a combination of TC-99 and blue dye or ICG. Despite its high DR and low false negative rate (FNR), it is inconvenient and costly.

### Injection Route

Injection routes include cervix and uterine corpus (47).

Cervical injection is the most common and simplest way. It is stable because of the rarity of cervical deformation caused by uterine fibroids, tumor infiltration or conization history (48). Anatomical studies have confirmed that cervical injection can penetrate into uterine vessels, isthmus, parametrial, and uterine body (15), while deep injection can reach para-aortic lymph nodes through pelvic funnel ligament (**Figure 1B**). The DR of pelvic SLN is higher using cervical injection as confirmed by large-scale studies (more than 100 patients), with a rate over 80% normally (49). However, the possibility of missing occult para-aortic lymph nodes (PAL) remains disputable. This may be compensated by rare incidence, ranging from 0.5% to 3.8% (47, 50, 51), of isolated para-aortic lymph node (IPL) metastasis, which is negative pelvic lymph node with positive PAL. Also, patients with any site lymph node metastases will receive adjuvant therapy, which theoretically eliminates potential metastatic lesions in para-aortic region (24). In brief, the main protocol for cervical injection is superficial injection (1–3 mm) with deep injection (1–2 cm or 3–4 cm) at 3- and 9-o’clock, or 3-, 6-, 9-, and 12- o’clock 2-, 4-, 8- and 10-o’clock points (48, 52) (**Figure 1C**).

Uterine corpus injection includes hysteroscopic or transvaginal ultrasound-guided peritumoral (subendometrial) injection (53) preoperatively and subserosal or myometrial injection intraoperatively. Hysteroscopic way can visualize the tumor directly and reflect the real lymphatic metastatic pathway, thus it seems a better method for the evaluation of para-aortic area. The DR ranged from 73% to 100%, the DR of PAL ranged between 13% and 56%, and the DR of IPL ranged from 3.4% to 20% (54–56). A multicenter RCT showed that hysteroscopic injection has a higher rate in identifying PAL (29% *vs.* 19.5, p=0.18) and IPL (5.8% *vs.* 0%) than cervical injection; however, there is no statistical difference (57). A recent retrospective analysis of 221 patients undergone hysteroscopic injection resulted in a 94.1%, 62.5%, and 2.7% DR, BDR, and IPL DR, which contributed to an 88.5% sensitivity and 96.5% NPV (58). However, the technique is complicated and not suitable for tumors with large size. Besides, the potential risk of tumor spreading through fallopian tubes is under concern (59). However, the risk of tubal leakage can be avoided by lower intrauterine pressure (<40 mm Hg) when performing hysteroscope and even tubal leakage may not result in tumor dissemination (60). The hysteroscopic way is usually injected around the tumor with 111MBq Tc 99m or 8 ml blue dye (60). Though subserosal injection at fundus is relatively easy, it remains difficult to show the parametrial lymphatic drainage, and most early ECs do not invade or penetrate to the serosa layer. Moreover, patients with uterine fibroids may cause uterine deformation, which made it difficult to inject. The reporting DR of subserosal injection varies from 73% to 95% (49). The injection site is generally at the midpoint of the uterine fundus, anterior wall, and posterior wall. Cervical isthmus and peritumoral regions can also be injected.

Overall, Cormier et al. conducted a systematic review of cervical injection in 1,102 cases and corpus injection in 300 cases, which led to a conclusion that the overall DR of cervical injection ranged from 62% to 100%, corpus injection varied from 73% to 95% (49), and the DR of PAL was 39%, 17%, and 2%, respectively, in fundus, deep cervix, and superficial cervix injection (49). Cervical injection is simpler, faster, and more effective, which is accepted and recognized as mandatory by worldwide gyn-oncologist in latest consensus and surgical assessment tool of SLNM in EC. It is noted that, in the consensus, cervical injection is obligatory, whereas hysteroscopic or myometrial injection is not suggested. Also, it recommends the utilization of ICG, although blue dye and Tc-99m are available (61).

## The Diagnostic Accuracy of SLNM

### Key Concepts

DR (49) refers to the percentage of patients with at least one SLN detected of all the patients tested. BDR refers to the proportion of patients with at least one SLN detected in each pelvic cavity to all the patients tested. False negative rate (FNR) refers to the proportion of patients with negative SLN but non-SLN positive to the total number of patients with SLN metastasis. Sensitivity is defined as the proportion of patients with positive SLN to the total number of patients with metastasis. NPV refers to the proportion of patients with SLN-negative and confirmed that no other lymph node metastasis to the total number of patients with SLN-negative. SLNM is supposed to show high sensitivity and low FNR.

### SLNM Shows Good Feasibility and Accuracy

The diagnostic value of SLNM requires the institution to perform LAD after SLNM and do pathologic evaluation of the lymph nodes resected by SLNM and LAD, respectively, to determine the abovementioned indicators. Researches have demonstrated high DR, sensitivity, and NPV in patients with early-stage EC using SLNM with pathologic ultra-staging. SLNM + LAD was performed in 125 patients with FIGO stage I-II EC by SENTI-ENDO multicenter research (62). The DR was 88.8%. The sensitivity, FNR, and NPV was 84%, 2.4%, and 97%, respectively. To improve the sensitivity and NPV, MSKCC proposed that, unilateral or bilateral LAD should be added if SLN map failure occurs in one side or both sides, all suspicious enlarged lymph nodes and peritoneal lesions should be removed, and ultra-staging pathology should be performed after operation. It is called MSKCC algorithm and is recommended in NCCN guideline. In a retrospective study involving 498 patients performed by Barlin et al. (63), the DR was 81%. After the SLN algorithm was applied, the FNR declined sharply from 14.9% to 1.9%, the sensitivity increased from 85.1% to 98.1%, and the NPV increased from 98.1% to 99.8%. When the MSKCC algorithm was retrospectively applied to 14 studies including SENTI-ENDO, NPV increased from 95% to 99.2% (47). The FIRES study (64) included 385 patients with EC from 19 surgeons in 10 institutions. The DR and BDR was 86% and 52%. The sensitivity, NPV and FNR was 97.2%, 99.6%, and 2.8%. However, these studies are mostly retrospective or prospective in nature, and there are no RCT yet.

The diagnostic value of SLN is thoroughly evaluated in several meta-analysis and systematic reviews, with the DR of SLN ranging from 80% to 100%, FNR varied between 0% and15%, and sensitivity ranged from 86 to 100%. The meta-analysis of 26 studies performed by Kang et al. (65) indicated that the DR and sensitivity was 78% and 93%, respectively. When learning curve deviation was considered, the DR and sensitivity with less than 30 patients were 82% and 88%, and those with more than 30 patients were 78% and 93%, respectively. Cormier et al. (49) conducted a systematic review of 17 studies, with the studies fewer than 30 patients excluded. The DR varied from 60% to 100%, and the DR exceeded 80% in subgroup of over 100 patients. After retrospective application of SLN algorithm, the sensitivity, NPV, and FNR was 95%, 99%, and 5% respectively. These results prove that surgeon experience and standard surgical procedures are favorable in improving diagnostic accuracy of SLN. Bodurtha et al. (66) published a meta-analysis of 4,915 patients in 55 studies. The DR and BDR was 81% and 50%. DR of PAL was 17%. The sensitivity and NPV was 96% and 99.7%, respectively. ICG and cervical injection could increase the DR (p < 0.05). The similar results were reached by Lin et al. (67) that ICG, cervical injection, and robotic-assisted surgery may improve the DR and sensitivity. While in a recent meta-analysis published by How et al. (68), with 5,348 patients and 48 studies included, the DR, BDR, and PAL DR was 87%, 61%, and 6%, respectively. It is noted that the study showed that SLNM failed to impair the diagnostic value in high-risk histology types, and compared with LAD, SLNM failed to affect survival outcome or increase recurrence risk.

### Factors Associated With Diagnostic Value

#### Surgeon Experience

Plenty of studies have demonstrated the learning curve effect. The accumulation of surgeon experience is associated with an increase in DR and sensitivity. Khoury et al. (69) compared the DR in early (2005–2007) and late (2008–2009) periods of MSKCC, which revealed an increase from 78% to 94%, suggested that the experience of more than 30 cases played an important role. Also, researchers from University of North Carolina finds 40 cases as a plateau of the learning curve for successful SLN mapping (70).

#### Tracer Type and Injection Site

ICG and cervical injection has gained worldwide acceptance for its ability in detecting SLN with a relatively high sensitivity. However, some researchers are working on combination dyes or special injection methods to improve the DR of both pelvic and para-aortic area and compromise the drawback of single method. Cabrera suggested adding Tc-99m to ICG for the increased BDR (69% *vs.* 41%, p = 0.012) and decreased empty node rate (0% *vs.* 4%, p = 0.032), which is known as a disadvantage to ICG alone (41). Our work shows an increased BDR in identifying SLNs when adding CNP to ICG comparing with CNP or ICG alone (45). Cervical reinjection when mapping failure occurs has been demonstrated as a feasible strategy to increase the DR and BDR of SLNM (71, 72). Eoh et al. (73) and Ruiz et al. (74) carried out “two-step method”, which was a combination of fundus injection and cervical injection of ICG, showing a relatively high DR in both pelvic and para-aortic regions. The overall DR of pelvic SLN and para-aortic SLN was 92.79% to 100%, and 86%, respectively. The sensitivity, specificity, and NPV ranged from 94.44% to 100%. Torne et al. developed transvaginal ultrasound-guided myometrial injection of radiotracer (TUMIR), presenting an 82.1% DR, 92.3% sensitivity, and 97.7% NPV (53).

#### Patient’s Condition

Age, obesity (BMI > 40), pelvic anatomical abnormality (vascular tortuosity), pelvic adhesions (history of operation and radiotherapy), and lymphatic vessel obstruction or destruction (tumor metastasis, deep myometrial infiltration, and endometrial inflammation), all could impact the DR of SLN (32, 37).

#### Pathology Examination

Some scholars believe that routine HE staining is possible to miss LVMD in SLN, which could be identified by immunohistochemistry staining (IHC) and serial section, also known as ultra-staging, which is discussed in later paragraphs.

#### Other Factors

At present, lymph vascular space invasion (LVSI), non-endometroid histology is seen as independent risk factors for failed mapping (75). The false negative SLN was more likely to appear in unilateral mapping failure patient. Higher SLN detection rate is also reported to be associated with tumor size and patient age, as well as tracer volume (76). However, the role of tumor size, depth of myometrial invasion, pathological type and grade, operation time, and scope, as well as LVSI are still lacking strong evidence. The other factor includes the surgical approach, like robotic or laparoscopic procedure. Cela et al reported 23 patients who underwent robotic-assisted surgery showing a 78.26% DR and 60.9% BDR (77). While, Chaowawanit et al summarized 76 patients with laparoscopic surgery and 33 patients with robotic approach. The result showed that laparoscopic procedure was superior than robotic in DR (97% *vs.* 83%, p = 0.046) and BDR (88% *vs.* 73%), whereas the two groups showed similar SLN detection and dissect time (78).

## The Therapeutic Safety of SLNM

Whether SLNM alone affects the long-term prognosis of patients with EC has been of great concern. Studies have been carried out to compare the oncologic outcome of SLNM-only *vs.* LAD without SLNM, or SLNM only *vs.* SLNM+LAD, or SLNM+LAD *vs.* LAD group, suggesting that SLNM failed to compromise survival outcome. Even though SLNM resects only a few lymph nodes, the overall DR of metastatic lesions in SLNM group is higher than regular LAD (79), which benefit accurate staging, thus guiding adjuvant therapy. In addition, SLNM can improve the quality of life for patients by minimizing operation complications (7, 8).

### SLNM Detected More Metastases Thus Facilitate Adjuvant Therapy

It is worth noting that even though SLNM may only remove two to four lymph nodes at a time, with a certain FNR and the risk of missing occult lymph nodes, the overall DR of metastatic lymph nodes is higher compared to conventional LAD (79). Leitao et al. (80) conducted a retrospective study on 507 EC patients. As indicated by the results, LAD rate decreased gradually and the number of removed lymph nodes was in decline accordingly (Y1 20; Y2 10; Y3 7; p < 0.001). However, there was no difference spotted in the detection of cases with lymph node metastasis found every year (Y1 7.0%, Y2 7.9%, Y3 7.5%, p = 1.0), so SLNM failed to reduce the diagnosis of stage IIIC. Despite this, it did reduce the need for LAD and the probability of surgical injury. In addition, Holloway et al. (79) found out that compared with LAD group (661 cases), SLN + LAD group (119 cases) showed a higher DR of metastases (30.3% *vs.* 14.7%, p < 0.001), more stage IIIC cases (30.2% *vs.* 14.5%, p < 0.001). SLN was the only metastasis in 50% of lymph node positive patients, and the FNR was 2.8%. SLN + LAD improved the DR of lymph node metastasis (OR3.29, p < 0.001). Raimond et al. (81) recruited 304 patients, and the incidence of lymph node metastasis in SLN was three times higher than in non-SLN (16.2% *vs.* 5.1%, p = 0.03). Among SLN positive, 8.1% were detected by ultra-staging. Furthermore, SLNM exerted no impact on recurrence-free survival (RFS). Buda et al. (82) found out that, in the early-stage patients, the DR of positive pelvic lymph nodes in SLN group (145 patients) was higher than in LAD group (657 patiens) (16.7% *vs.* 7.3%; p = 0.002), including 80 type II EC, and there was no difference observed in 3-year RFS and mortality between the two groups.

The improved detection rate of metastases probably attributes to the application of ultra-staging pathology, which help find previously neglected metastases, and the identification of lymph nodes located outside the routine lymph node dissection area. As revealed by the FIRES studies, 17% of lymph node-positive patients were found in non-traditional sites (presacral, parametrial areas, and deep iliac) (64). Therefore, the improvement to DR of metastatic lesions may mitigate the false negative consequences of SLN.

### SLNM Did Not Impair Survival Outcome

Although long-term follow-up studies and RCTs for the comparison of survival outcome between SLNM and LAD are lacking, current results showed promising results that SLNM did not compromise the survival prognosis of EC patients (47).

In the SENTI-ENDO study conducted by Darai et al. (83), the outcomes of 125 stage I-II EC patients were assessed. There was no difference observed in recurrence rate (12.6% *vs.* 28.6%; p = 0.23) and RFS between successful SLN detection group and failed group. There was no difference in RFS between lymph node metastasis group and non-metastasis group (p = 0.23). However, the adjuvant therapy in the study was not standardized and it is difficult to validate the accurate survival effect of SLNM. Eriksson et al. (84) applied two lymph node dissection methods to patients with low-risk EC in MSKCC and Mayo Clinic, respectively. MSKCC applied SLN algorithm (642 cases), and Mayo Clinic applied SLAD (493 cases). The results indicated that the DR of metastasis was higher in SLN group. The pelvic lymph node metastasis rate (including LVMD) was 5.1% and 2.6% (p=0.03), respectively, while the PAL metastasis rate was 0.8% and 1.0% (p=0.75), respectively. There was no difference in 3-year disease-free survival (DFS) (94.9% *vs.* 96.8%), despite that the adjuvant treatment rate in SLNM group was higher than in SLAD group (27.1% *vs.* 10.8%; p < 0.001). Similar studies have been carried out in two Italian institutions (82) and totally 802 patients with early-stage EC were included. After 30-month median follow-up, there was no difference observed in DFS (p=0.396) and OS (p=0.394) between SLNM group and SLAD group. How et al. (85) recruited 275 SLNM + LAD patients and 197 LAD patients for study, which revealed that in clinical stage I patients, there was neither difference in the incidence and type of adjuvant therapy between the two groups, nor difference in RFS. The recurrence rate of pelvic wall in SLNM + LAD group was lower (31% *vs.* 71%). The former exhibited a reduced pelvic wall recurrence rate by 68% (HR 0.32, p=0.007). The authors suggested that SLNM may be superior to LAD due to the removal of lymph nodes at a higher risk of metastasis. However, this study can only prove that SLNM + LAD reduced the recurrence rate compared with LAD alone, for which it can hardly prove the advantages of SLNM alone. Imboden et al. (7) concluded that SLNM offered a considerable balance between oncologic safety and perioperative morbidity in 275 early-stage, G1 or G2 patients, especially for LVSI-positive. As shown in a meta-analysis recently published by Bogani et al. (2), compared with LAD, SLNM exhibited no difference in recurrence rate and PAL metastasis. In addition, A cohort study with 5546 patients published by Polcher et al. (86) indicated that LAD failed to improve DFS or OS compared with SLNM. On the contrary, it resulted in more complications in the high-risk histology type. The most recent multi-institutional retrospective study performed by Bogani et al. (87) compared the long-term oncologic results of SLNM, SLNM+LAD and LAD. The results found that there was no statistical difference between the three strategies in DFS (p=0.570) and OS (p=0.911); moreover, the survival outcome was similar in low risk, intermediate risk, and high-risk group. Kogan et al (88) compared 193 EC patients with LAD and 250 patients with SLN+LAD. They found that SLN may improve the oncologic outcome with a more favorable 6-year OS (HR 0.5, 95% CI 0.3-0.8, p = 0.004) and PFS (HR 0.6, 95% CI 0.4-0.9, p = 0.03). Also, SLN seemed to reduce the risk of recurrence in pelvis or lymph node region with a 6-year RFS of 95% compared to 90% (p=0.04) in LAD only group. Recently, Jayot et al. from France analyzed 248 EC patients between 2007 and 2018 undergone SLN procedure, as a result, the 3-year OS was 99% and 3-year RFS was 92% (89).

### SLNM Reduced Intraoperative and Postoperative Complications

The most common complication of LAD was lymphedema, followed by lymph cysts, vascular and nerve injury, blood loss, and prolonged operation, etc. It seems that these risks can be reduced with the application of SLNM. Accorsi et al. (90) found that compared with TH, SLNM did not increases the incidence of intraoperative complications (p=1.0) and postoperative complications (p=0.782). While LAD laid more risk on intraoperative complications (HR, 14.25;95% CI, 1.85–19.63), postoperative complications (HR, 3.11; 95% CI, 1.62–5.98), and lower-extremity lymph edema (HR, 8.14; 95% CI, 1.01–65.27). Geppert et al. (91) drew comparison of the perioperative outcomes for TH + BSO, TH + BSO + SLN, and TH + BSO + LAD groups. The average operation time of SLN group and LAD group was found to be extended by 33 and 91 min, respectively. The incidence of lower limb lymphedema in SLN group was significantly lower than in LAD group (1.3% *vs.* 18.1%; p=0.0003). The same result was shown by Persson et al. (72, 92), in which SLNM reduced the risk of lower extremity lymphoedema by 14 times. In addition, Liu et al. (93) found that SLNM group significantly reduced the incidence of postoperative complications (5.2% *vs.* 13%; p=0.04), decreased intraoperative blood loss (56 ml *vs.* 80 ml; p=0.004), and shortened the operation time (137 min *vs.* 181 min; p <0.0001), meanwhile, the average number of lymph nodes dissected was significantly decreased (4 *vs.* 15; p <0.0001). When comes to lymphedema and lymph cyst, MSKCC concluded that SLN mapping was an independent factor in reducing patient reported lower-extremity lymphedema, while high BMI and adjuvant EBRT were associated with increased lymphedema (94). While another research stated that systemic LAD was the only factor that associated with the presence of lymphocele, the number of dissected nodes showed no impact. Compared with SLN+LAD group, SLN only group significantly decreased lymphocele rate from 14.1% to 3.4% (p=0.009) (95). Mayo Clinic analyzed 378 patients and found that SLN may significantly decrease the risk of lymphedema compared with LAD (26.0% *vs.* 49.4%, p<0.001) (96). Several meta-analyses included current retrospective and prospective studies presented similar conclusions, which was SLN resulted in less blood loss, lymphedema, and other complications, meanwhile, SLN detected more pelvic metastasis (97, 98). These results may indicate that SLNM is able to minimize the surgical risk and reduce the complications with no survival detriment in EC, which is of great value to improve the quality of life.

## The Application of SLNM in Early-Stage High-Risk EC

Nowadays, it is trending to carry on SLNM in early-stage high-risk EC, including high-risk histology (G3 endometroid, serous carcinoma, clear cell carcinoma, and carcinosarcoma), deep myometrial invasion, cervical involvement, and LVSI (+). Some institutions are making attempt to apply SLNM as routine surgical staging in all EC patients, except for patients with suspected lymph-node metastasis or failed mapping. Previous studies are typically performed on early-stage EC patients with mostly patients with lower-risk of recurrence and fewer higher-risk included. Recent studies attempted to evaluate the diagnostic accuracy and oncologic safety of SLNM in early-stage high-risk patients only. Though there are no RCTs published yet, existing evidence indicates that SLNM may be also efficient and safe in high-risk group, MSKCC has already established SLNM as a routine procedure for all candidate patients, including serous and carcinosarcoma type. However, it is essential to choose appropriate indication and strictly comply with SLN algorithm when using SLNM in high-risk patients (10).

The potential diagnostic value of the SLNM in high-risk patients has been proven in recent years. The DR ranges from 73% to 100%, the BDR varies from 56% to 95%, and NPV ranges from 93% to 100% (5, 42, 53, 72, 92, 99–107). Both SENTI-ENDO study and FIRES studies include low-risk and high-risk EC and present high DR and NPV (64, 83). There have been studies only including high-risk patients to evaluate the diagnostic value (**Table 1**). Frumovitz et al. performed study on 18 high-risk EC patients in 2007. The SLN DR was merely 45% (108), which may be the result of technique and surgeon experience. Then Torne et al. operated SLNM+LAD+PALAD on 74 high-risk patients in 2013, while the DR, sensitivity, and NPV were 74.3%, 92.3%, and 97.7% (53), respectively. Subsequently, in 2015, Farghali et al. showed a 73.1% DR, 94.4% sensitivity, and 100% specificity in 93 high-risk patients (100). In 2016, Ehrisman et al. demonstrated an increase from 92.3% to 100% in NPV by applying SLN algorithm to 36 high-risk EC patients (101). In 2017, plenty of constructive research results were obtained. For example, Soliman et al. performed SLNM+LAD+PALAD under 123 high-risk patients. Nineteen percent of the patients diagnosed with stage III exhibited DR, sensitivity, and FNR of 89%, 95%, and 4.3% (103), respectively. Baiocchi et al. included 236 high-risk EC patients. As a result, the SLN arm has a sensitivity of 90%, an NPV of 95.7%, and an FNR of 4.3%. Besides, the positive lymph node DR is significantly increased in SLN group compared with LAD group (26.7% vs. 14.3%, p=0.02) (106). In the same year, a multi-institutional research was conducted by Touhami et al., who performed SLNM+LAD+/−PALAD in 128 high-risk EC patients (including undifferentiated type). They found out that the sensitivity and NPV of SLNM were 95.8% and 98.2%, respectively (104). Furthermore, in 2018, Papadia et al. conducted analysis of 42 high-risk patients (including neuroendocrine cancer). They reported that the DR and BDR of SLN were 100% and 90.5%, respectively. Excitingly, the sensitivity and NPV were both 100% (42). Sweden teams performed robotic surgery on 257 stage I-II high-risk EC patients, resulting in a sensitivity of 100% and a NPV of 100%. The BDR was as high as 95%, and no adverse effect occurred (72). Wang et al. recently published their data and found a DR of 86.7% and FNR, NPV and sensitivity was 11.8%, 97.3% and 88.2% respectively. When considering SLN algorithm and surgical experience (over 30 cases), the FNR and NPV increased (105). Thus, SLNM seems to be feasible in high-risk context with an acceptable DR and diagnostic value. However, Ye et al analyzed 131 patients using ICG and SLNM followed by LAD (112). The sensitivity and NPV were unexpectedly as low as 20% and 83.3%, with a surprisingly high FNR of 80%. The author considered the risk of missing IPL of SLNM in high-risk patients may be the reason, a large-scale multicenter study was needed to clarify the result.

**Table 1:** The diagnostic value of SLNM in high-risk EC.

Author	Year of publish	Country	Study type	Study period	Number of pts	Histology	SLN method (dye and injection site)	Surgery approach	Overall DR	BDR	PASDR	Sensitivity	NPV	FNR
Burke et al. (30)	1996	USA	pilot	NA	15	EEC(G2,G3), CC, USC	BD; subserosal, myometrium	Lpt	67%	NA	NA	66.70%	87.50%	33.30%
Frumovitz et al. (108)	2007	USA	pro	2002-2004	18	EEC(G2,G3), CC, USC	BD, Tc; Fundus	Lps	45.00%	5.56%	22.22%	NA	NA	NA
Torne et al. (53)	2013	Spain	pro	2006.03-2011.03	74	EEC(G3),CC,USC,DM,CI	Tc; TUMIR	Lps	74.30%	14.00%	45.40%	92.30%	97.70%	7.70%
Perissinotti et al. (99)	2013	Spain	pro	2007.06-2010.12	44	EEC(G3),CC,USC,USM,DM	Tc; TUMIR	Lps	73.00%	NA	NA	NA	NA	NA
Farghali et al. (100)	2015	Egypt	pro	2007.05 -2011.05	93	EEC(G2,G3), CC, USC	BD; subserosal, myometrium	Lpt	73.10%	40.86%	0.00%	94.40%	98.90%	5.88%
Valha et al. (109)	2015	Czech	pro	2012.06-2014.02	18	stage I-II, intermediate and high-risk	BD; subserosal	Lpt	88.89%	NA	50.00%	NA	NA	NA
Ehrisman et al. (101)	2016	USA	retro	2012.08-2015.06	36	EEC(G3),CC,USC,CSM	BD,ICG;cervical	Lps,Rb	83.00%	56.00%	3.00%	77.80%	92.30%	22.22%
Baiocchi et al. (106)	2017	Spain	retro	2007.06-2017.02	236(75 SLN+LAD; 161 LAD)	EEC(G3),CC,USC,CSM,DM,LVSI	BD; cervical	Lps,Rb,Lpt	85.30%	60.00%	1.50%	90.90%	95.7%,	10.00%
Tanner et al.J (110)	2017	USA	retro	2012.12- 2015.12	52	EEC(G3),CC,USC,CSM	BD,ICG;cervical	Lps,Rb	86.00%	59.60%	9.00%	77.80%	94.70%	22.20%
Soliman, PT (103)	2017	USA	pro	2013.04- 2016.05	101	EEC(G3),CC,USC,CSM,DM,CI	ICG, BD, BD+Tc; cervical	Lps,Rb,Lpt	89.00%	58.00%	2.00%	95.80%	98.20%	5.00%
Touhami et al. (104).	2017	Canada	retro	2010.11- 2016.11	128	EEC(G3),CC,USC,CSM,undifferentiated	BD, Tc, ICG; cervical	Lps,Rb,Lpt	89.80%	63.20%	5.00%	97.43%	98.80%	2.56%
Ducie et al. (107)	2017	USA	retro	2006–2013	120	EEC+any grade+DM;USC, CC	BD, ICG; cervical	NA	NA	NA	NA	96.40%	98.90%	3.60%
Buda et al. (111)	2018	Italy, Switzerland	retro	NA	171	ESMO high-intermediate and high risk	ICG, Tc+BD; cervical	NA	98.00%	80.1%(ICG); 65.7%(BD,Tc)	NA	85.2%; 91.2% for algorithm	93.4%;96% for algorithm	14.7%;8.8% for algorithm
Papadia et al. (42)	2018	Switzerland	retro	2012.12 - 2017.07	42	EEC(G3),CC,USC,CSM,NEC	ICG; cervical	Lps	100%	90.50%	NA	90%;100% for algorithm	97%;100% for algorithm	10%;0% for algorithm
Persson et al. (72)	2019	Sweden	pro	2014.06-2018.05	257	EEC(G3),non-EEC, DM, CI, non-diploid cell	ICG; cervical+/-reinjection	Rb	NA	82%; 94.8% after reinjection	NA	98%; 100% for algorithm	99.5%;100% for algorithm	3.7%;0% for algorithm
Wang et al. (105)	2019	China	retro	2016.08-2018.08	98	EEC(G3),CC,USC,CSM,EEC(G1,G2) +DM,CI	ICG; cervical	NA	95.92%	77.60%	NA	88.2%; 90.9% for algorithm	97.47%; 97.30% for algorithm	11.8%; 9.1% for algorithm
Ye et al. (112)	2019	China	pro	2016.07-2018.07	131 pts with 25 high-risk	EEC(G3),CC,USC,CSM,undifferentiated	ICG; cervical	Lps	100%	72%	NA	20%	83.30%	80%
Angeles et al. (76)	2020	Spain	pro	2006.03-2017.03	123	intermediate and high-risk EC	TUMIR	NA	70.70%	NA	NA	NA	NA	NA
Taskin et al. (113)	2020	Turkey	retro	2017.05-2018.11	38	high-risk (Mayo criteria)	ICG; cervical	Lps,Rb,Lpt	84.21%	68.40%	NA	80%	93.40%	NA

pts, patients; SLN, sentinel lymph node; LAD, lymphadenectomy; DR, detection rate; BDR, bilateral detection rate; PAS, para-aortic SLN; NPV, negative predictive value; FNR, false negative rate; NA, not applicable; EEC, endometrioid endometrial cancer; G, grade; CC, clear cell carcinoma; USC, uterine serous carcinoma; CSM, carcinosarcoma; DM, deep myometrial invasion; CI, cervical involvement; BD, blue dye; Tc, Technetium-99; TUMIR, transvaginal ultrasound-guided myometrial injection of radiotracer; Lpt, laparotomy; Lps, laparoscopic; Rb, robotic surgery; pro, prospective; retro, retrospective.

Moreover, prospective and retrospective studies indicated that SLNM appears to have no negative impact on oncologic outcomes in high-risk EC patients (**Table 2**). MSKCC conducted a retrospective analysis of 136 patients with uterine carcinosarcoma in 2016 (114). The result showed that there was no difference in PFS (23 *vs.* 23.2 months; p=0.7) and detection of metastatic lymph nodes (p=0.2) between SLN group and LAD group. Local recurrence rate was 15% in SLN cohort and 24% in LAD cohort, which is consistent with previous study conducted by How et al. (85). Subsequently, in 2017, MSKCC retrospectively evaluated 248 patients with uterine serous carcinoma. No difference was observed either in the diagnosis of stage III/IV, adjuvant therapy rate, and 2-year PFS between SLN group and LAD group. However, the incidence of local recurrence was 9.7% and 9.1% in SLN group and LAD group (115). The exact effect of SLNM and related adjuvant therapy on local recurrence control needs to be further investigated. The same histology type was further and thoroughly reviewed by MSKCC in a recent paper published by Basaran et al. (118). This time, they carefully categorized uterine serous carcinoma patients in January 1996 to December 31, 2017 into SLN only group (79) and LAD without SLN group (166). The two cohorts showed no survival difference in stage I to III uterine serous carcinoma as they yielded similar detection of nodal metastasis. Also, PALND did not show any survival benefit on OS. Moreover, MSKCC and Mayo Clinic investigated 176 deeply invasive endometrioid EC in 2018. When other factors were balanced, the PFS, OS, and recurrence rate exhibited no difference (117). Additionally, in 2018, Buda et al. reported an Italian multicenter study, which included 266 high-risk patients. The 3-year DFS and OS showed no difference in SLN group and SLAD group (116). In the same year, Buda et al. published data obtained from Italian and Swedish multicenter of 171 high-risk EC patients. The 5-year DFS indicated no difference among SLN group and SLAD group (111). The impact of SLNM on clear cell carcinoma was investigated by Mayo Clinic and MSKCC (119). The researcher included early stage serous or clear cell endometrial carcinoma with any degree of myometrial invasion. The results showed that SLNM cohort (118 patients) did not increase lymphatic recurrence and exhibit a similar OS (88% *vs.* 77%, p=0.06) with LAD cohort (96). However, in node-negative cases, SLNM group may be associated with decreased RFS (73% *vs.* 91%, p=0.05), despite the majority of SLNM patients received chemotherapy (84% *vs.* 40%, p < 0.001). Most recently, Bogani et al. compared SLN alone and SLN followed by LAD (121) in 196 high-risk patients (121). The two groups showed no difference in DFS (p = 0.416) and OS (p = 0.940) despite that LAD removes more positive nodes.

**Table 2:** The oncologic outcomes of SLNM in high-risk EC.

Author	Year of publication	Country	Study type	Time period	Patient group (N)	Histology	LN positive rate	p value	DFS	p value	OS	p value	Distant recurrence rate	p value
Schiavone et al. (114)	2016	USA	retro	1998.01-2014.08	SLN-A(48)	USM	22.90%	p=0.4	23m(2y)	p=0.7	NA		70%	NA
					LAD(88)		21.59%		23.2m		NA		74%	
Ducie et al. (107)	2017	USA	retro	SLN (2006–2013)	SLN-A(120)	EEC: any grade, MI>50%; USC/CC, any MI.	21.70%	p=0.68	NA		NA		NA	
				LAD (2004–2008)	SLAD(103)		19.40%		NA		NA		NA	
Schiavone et al. (115)	2017	USA	retro	2005.01-2015.07	SLN-A(153)	USC	31%	p=0.3	77%	p=0.3	NA		15.03%	NA
					LAD(95)		38%		71%		NA		23.16%	
Baiocchi et al. (106)	2017	Spain	retro	SLN (2007.06-2017.02)	SLN+LAD(75)	EEC(G3), CC, USC, CSM, DM, LVSI	26.70%	p=0.02	NA		NA		NA	
				LAD (2012.11-2017.02)	LAD(161)		14.30%		NA		NA		NA	
Buda et al. (111)	2018	Italy, Switzerland	retro	NA	SLN-A(66)	High-intermediate and high-risk	27.30%	p=0.297	79.20%	p=0.831	NA		0	NA
					SLN+SLAD(105)		32.40%		81.60%		NA		0.95%	
Buda et al. (116)	2018	Italy	retro	2010.10-2014.02	SLN(61)	High-intermediate and high-risk	16.70%	p=0.002	HR: 0.92(3y)	p=0.646	HR: 0.92(3y)	p=0.675	NA	
					LAD(139)		7.30%						NA	
Schlappe et al. (117)	2018	USA		SLN (2005–2013)	SLN-A(82)	DM EEC	33.30%	p=0.005	adjusted HR:0.87	NA	adjusted HR:2.54	NA	20.80%	NA
				LND (2004–2008)	LAD(94)		14.80%						14.90%	
Basaran et al. (118)	2020	USA	retro	1996.01-2017.12	SLN alone(79)	USC	26.50%	p=0.6	58.8%(2y)	p=0.478	89.1%(2y)	p=0.9	36.7%^※^	p=0.524
					LND without SLN (166)		29.50%		64.9%(2y)		83.9%(2y)		40.9%^※^	
Schlappe et al. (119)	2020	USA	retro	2006- 2013	SLN(118)	USC/CC with any MI	21.70%	p=0.83	68.9%(3y)	p=0.32	87.9%(3y)	p=0.06	NA	
				2004- 2008	LND(96)		20.50%		80.3%(3y)		76.8%(3y)			
Nasioudis et al. (120)	2020	USA	retro	2012-2015	SLN(460)	EEC(G3) and non-EEC	10.5%	p=0.10	NA		84.3%(3y)	p=0.86	NA	
					LND(920)		13.30%	NA	NA		86.8%(3y)			
Bagoni et al. (121)	2021	Italy	retro	2009.01-2019.12	SLN(50)	EEC(G3) with MI >50% and non-EEC	28%		NA	p=0.416	NA	p=0.940	16%	0.413
					SLN+LAD(146)		23.20%		NA		NA		12%	

^※^The data refers to all types of recurrence.

N, number; LN, lymph node; DFS, disease-free survival; OS, overall survival; pro, prospective; retro, retrospective; SLN-A, SLN-algorithm; LAD, lymphadenectomy; EEC, endometrioid endometrial cancer; G, grade; CC, clear cell carcinoma; USC, uterine serous carcinoma; CSM, carcinosarcoma; MI, myometrial invasion; DM, deep myometrial invasion; LVSI, lympho-vascular invasion; m, months; y, year; NA, not applicable; HR, hazard ratio.

However, it is noted that only a few intuitions perform SLN-algorithm only in the SLN cohort for the comparison study, whereas others are more likely to perform LAD followed by SLNM, thus, the results are rather a comparison between SLN+LAD and LAD, which make the survival results less convincing and more complicated. The role of backup LAD for high-risk cases remains areas of investigation. Also, there are studies addressing the problem and comparing the oncologic outcomes between SLN and more extensive LAD with or without SLN, preliminary results suggested that there are no difference in these approaches (87, 118).

These results may indicate that the application of SLNM in high-risk EC patients is as efficient and safe as in the lower-risk type, for accurate staging, thus guiding adjuvant therapy, suggesting SLN may be an optimal choice for high-risk patients. However, the effect is attributed to the adjuvant therapy based on lymph node status or eradication of lymph node metastases directly is unclear, since earlier studies did show a survival benefit for patients did systemic LAD with an average of 12 lymph nodes moved (122), and the current favorable studies are limited by its prospective or retrospective nature. Lack of RCTs, long-term follow-up studies, standardized SLNM technique, and ultra-staging protocol, as well as adjuvant therapy are the primary concern. In an ideal clinical research, the patients should be randomly assigned into SLNM arm or LAD arm, and receive standard post-operative adjuvant therapy according to stage information (40). It is plausible to add LAD, particularly PALAD, in high-risk group before high-quality evidence is published.

## Current Application of SLNM

SLNM is gaining widespread utilization for staging in EC. It was first written in the NCCN guideline since 2014. And for now, FIGO and NCCN all support the utilization of SLNM in apparent uterine-confined EC despite lack of RCTs. Studies have proven that SLNM with ultra-staging may be effective in providing prognostic information for regional lymph node, choosing adjuvant therapy, and reducing operation complications.

NCCN recommends the application of SLNM in EC patients with lesions apparently confined to the uterine cavity without any extra-uterine metastases on imaging examination. Meantime, NCCN also permits the potential use of SLNM in early-stage high-risk EC patients like serous carcinoma, clear cell carcinoma and carcinosarcoma (123). Surgeons must strictly follow the technical details and SLN algorithm in operation, including superficial and deep injection of cervix, thorough evaluation of abdominal and pelvic cavity, resection of all SLN and suspicious enlarged lymph nodes, additional LAD on unmapped side when SLN mapping failure occurs and ultra-staging pathology is performed in combination with routine H&E. Whether to perform PALAD is at the discretion of the surgeon (48). While in the latest consensus and surgical assessment tool, which aims to standardize the surgical technique and quality of SLNM in EC, it also recommends cervical injection of ICG, however, when mapping failure occurs, it points out 4 choices: waiting and turning to contralateral side, exploring the uncommon regions like presacral, common iliac or para-aortic area, re-injection of tracer, or performing side specific LAD (61).

Moreover, the application of FS of SLN is in debate due to the low sensitivity, expensive price, and the propensity to neglect LVMD (10, 26). However, Tanner et al. (110) argued that it was plausible to add FS when SLN map failure occurs, which was called “reflux FS”, as it could reduce the need for LAD based on uterine factors by decreasing the rate from 18.6% to 7.1%. Besides, they recommended a direct LAD instead a reflux FS for high-risk EC. Similar results were obtained by Sinno et al. (27) and Altin et al. (124). Thus, NCCN guideline suggests that secondary SLAD may be considered in the cases of failed SLN mapping (125). In addition, Renz et al. (126) from Stanford University and Bellaminutti et al. (127) from Switzerland found that adding intraoperative FS to SLN can find micrometastases with a good accuracy, and NPV, thus, may identify patients who are in need for a systemic LAD for dissecting additional lymph node metastases.

At present, the application of SLNM is gradually expanding, and more than 70% of patients may be suitable for SLNM (50). Recent surveys from ESGO and SGO confirmed that 50.2% (128) of European gynecological oncologists and 82.7% (129) of USA gynecologic oncologists adopted SLN in EC. In low-risk patients, who usually do not have to perform LAD, there are 2.4% lymph node metastatic potential (5), especially in LVSI positive patients (7). Additionally, LVMD is more likely to occur in low-risk patients (130). SLNM can remove fewer lymph nodes with sufficient staging information supporting adjuvant therapy, and will not cause the possibility of post-operative complications to increase compared with hysterectomy alone (7). In high-risk patients who should undergo LAD, approximately 80% (5, 107) of them do not have lymph node metastasis. Moreover, it is difficult for obese patients and patients with severe internal complications to tolerate LAD. Also, adjuvant therapy can eliminate obscured metastases that are not found in surgery theoretically as supported by many clinical trails (131), which showed that concurrent chemoradiotherapy can significantly extend PFS and OS in advanced EC patients. Moreover, SLNM improves the detection of metastases by identifying LVMD with assistance of ultra-staging and identifying lymph nodes in non-regular region, which is significant for accurate staging and choosing adjuvant therapy. Despite lack of RCTs and long-term follow-up studies, existing evidence advocate the utilization of SLNM in uterine-confined EC even in high-risk histology because of sufficient detection rate of SLN and nodal metastases, and similar survival outcome compared with conventional LAD. It is worth expecting long-term survival outcome, cost-performance, and complication incidence of SLNM in early-stage EC patients in ongoing randomized clinical trials.

## Controversial Issues

### Pathological Ultra-Staging and LVMD

Pathological assessment methods for lymph nodes include H&E and IHC staining. Ultra-staging is a combination of serial section and IHC (anti keratin AE1:AE3) to identify the LVMD (10, 15, 66). The standard set by SGO about LVMD is based on breast cancer guidelines published by AJCC (132): macro-metastasis (> 2 mm); low-volume metastases (LVM), including micro-metastasis (MM) (0.2−2 mm) and isolated tumor cells (ITCs) (< 0.2 mm). AJCC (133) set term pN0 (i+) for ITCs and pN1mi for MM in breast cancer. In NCCN guideline, pN0 (i+) is set for ITCs in EC patients (125). A more accurate staging may be needed to guide further personalized adjuvant therapy and evaluate prognosis.

**Figure 2:**
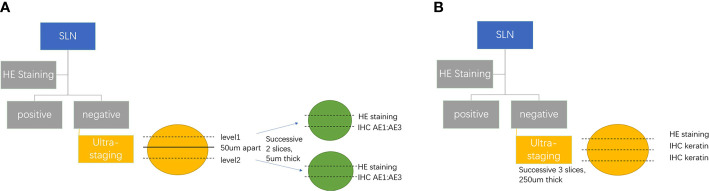
**(A)** MSKCC SLN ultra-staging protocol. **(B)** M.D. Anderson Cancer Center SLN ultra-staging protocol.

There is no standardized protocol for ultra-staging yet. MSKCC (134) divides H&E-negative SLN into two levels (50 μm apart). Then, if the previous one remains H&E negative, two consecutive 5-µm thick sections are sliced at every level, one for H&E and the other for IHC (**Figure 2A**). M.D. Anderson Cancer Center (135) cut three serial 250-μm-thick sections for lymph node which has a negative H&E, with one repeating H&E. If it is still negative, the other two slices undergo IHC (**Figure 2B**). As indicated by reports, there is no difference between two kinds ultra-staging on the detection of SLN metastases for both high-risk and low-risk EC patients (26, 136).

The incidence of LVMD varies approximately from 3.8% to 19.7% (10, 62, 79, 130, 134, 137, 138) However, the LVMD detected by ultra-staging accounts for almost 50% of all lymph node metastases. The risk factors related with LVMD are LVSI, unfavorable histology, myometrial invasion, and so on. Yabushita et al. (139) figured out the relevance between LVSI and the positive expression of keratin in IHC staining. The author stated that keratin positive is the independent risk factor for recurrence. Todo et al analyzed 61 EC patients with intermediate risk for recurrence (140). The results showed a 14.8% incidence of LVMD and deep myometrial invasion was significantly associated with ITC/MM (p=0.028). Bogoni et al. (130) hold that LVMD is more likely to be detected in low-risk patients. However, research done by Mueller et al. concluded that ITC incidence increased with depth of myoinvasion. Twenty-five percent of deeply invasive G1/G2 and 18% of deeply invasive G3 tumors had ITCs compared to a rate lower than 1% in non-invasive endometroid EC patients. When coming to non-invasive serous type, the incidence for ITC goes up to 10% (141).

Though the clinical significance of LVMD remains under investigation, more stage IIIc patients are diagnosed by ultra-staging and 5% to 15% patients face upstaging (134). Whether MM or ITC need adjuvant therapy and indicate better or worse prognosis are conflicting. Recent data tend to consider patients with MM for a following adjuvant therapy, whereas patients with ITCs do not. Todo et al. (140) concluded that LVMD was an independent risk factor for extra-pelvic recurrence. Compared to node-negative patients, a noticeable 20% decrease was observed in 8-year OS and PFS in LVMD patients. However, no statistical difference was calculated. MSKCC (142) reported a large cohort study that 5.2% patients had LVMD and 5.6% patients found macrometastases. As a result, the LVMD group shows a significant increase in 3 year-RFS compared with the macro-metastases group (86 vs. 71%, p <0.001), as most LVMD receive adjuvant therapy. Plante et al. (143) published a single center prospective study involving 519 EC patients. The 3-year PFS was 95.5% for ITCs, which was similar to MM (85.5%) and lymph node negative (87.6%) and much better than macro-metastases (58.5%). Brugger et al. (50) found out that patients with ITC and MM received more adjuvant therapy and presented much better oncologic outcomes. A recent review published by Bogani et al. believed that the patients with MM detected in SLN should receive adjuvant therapy, whereas whether ITC undergoes adjuvant therapy depends on uterine factors (130). The similar conclusion was reached by Goebe et al, in which they sent 155 SLN negative patients tissue slides into IHC staining retrospectively (144). Even though 13.5% of SLN negative patients found ITCs, no recurrence was found in patients had previously undetected ITCs without receiving adjuvant therapy as well, suggesting that ITCs may not be relevant to recurrence risk. However, Sawicki et al. (145) stated that LVMD are independent of histology type, myometrial invasion, LVSI and cervical invasion and they does not affect prognosis. It is noted that in breast cancer, though LVMD is recorded in staging, they do not influence the treatment decision for they do not change survival (126).

In addition, ultra-staging improved the detection of nodal metastasis to two times compared with normal H&E, and interestingly half of positive lymph nodes are SLN (79, 81). SLN may have an advantage in identifying LVMD. Niikura et al. (146) obtained a 5% of LVMD in SLN, compared to merely 0.3% in non-SLN. FIRES study (64)also indicated that SLN is more likely to identify metastases than non-SLN (5% *vs.* 1%, p=0.0001). Moreover, compared with traditional LAD, which removes over 20 lymph nodes, SLNM, which removes less than four lymph nodes in most papers, reduces the workload and makes ultra-staging more feasible for pathologists. SLNM permits a possibility that pathologist could pay attention to fewer lymph nodes. However, it is noted that most institutions only perform ultra-staging on SLN but non-SLN due to many factors, which may underestimate the incidence of LVMD in non-SLN.

Nevertheless, ultra-staging owns such limits, which is time-consuming, that cannot be done intraoperatively, whereas intraoperative FS seems to be low sensitivity in identifying LVMD and the discrepancy between pathologists and institutions. OSNA, which is one-step nucleic acid amplification, comes to the researchers’ eyes. It is a molecular-based method for the detection of metastatic lymph nodes in breast cancer or colorectal cancer patients using CK19 as a single marker. Mounting evidence has demonstrated good sensitivity and specificity of OSNA in identifying positive nodes, especially micro-metastasis, in endometrial cancer (147–150). Compared with ultra-staging, OSNA is much faster thus can be done intraoperatively; moreover, it identified more SLN involvement, resulting in 20.69% of patients upstaged as FIGO stage III (150). The technique is autonomous and quantifiable, which saves pathologist’s work and makes results more comparable and less variable (151). Also, the use of the entire lymph node avoids insufficient analysis of pathology, thus increasing the identification of metastatic lesions. However, one limit is the risk of false-positive cases as CK19 can also be expressed in normal endometrium (152), so developing new specific markers may be necessary. Also, the method needs an entire node which makes morphologic observation of metastatic features unachievable (147), as well as future research for other molecular testing (153). Moreover, the cost is almost 10 times higher than the current pathology examination (147) and the best cutoff value for identifying macro-metastasis and LVMD, as well as predicting non-SLN involvement in EC may need further investigation (154).

### Aortic Lymph Node Dissection

Anatomical study has proven that EC can directly metastasize into PAL through pelvic-infundibular ligament pathway. Currently, the dissection of para-aortic area is left to the surgeon’s decision based on NCCN SLN algorithm. The possibility of missing occult PAL metastasis, especially IPL metastasis, is one of the primary concerns of SLNM. But existing evidence shown that the incidence of IPL metastasis is rare with approximately 0.5% to 3.8% (49, 51). Chiang et al. (155) summarized 18 papers and concluded that the incidence of IPL metastasis is as low as 1.7%. Kumar et al. (21) demonstrated that lymph node metastatic rate for para-aortic region and pelvic cavity is similar (12% *vs.* 17%). When pelvic lymph nodes are positive, 51% have PAL metastases. Whereas when pelvic lymph nodes are negative, PAL metastases, namely IPL metastasis, are found in 3% patients. Usually, patients with positive pelvic lymph nodes would receive adjuvant therapy, which could eliminate the possible aortic lesions in theory, for SLNM with ultra-staging has an excellent ability to detect pelvic metastasis with high sensitivity and NPV. Also, researchers have developed strategies like “dual site injection (156)” or “reinjection (72)” to increase the detection of aortic SLN to reduce FNR. Researchers from Korea showed that a sequential administration of bilateral uterine cornus injection of ICG followed by cervical injection, improved the para-aortic SLN detection rate from 5.7% to 38.2% in upper para-aortic area (p<0.001) and 18.7% to 67.1% in lower para-aortic area (p<0.001), which in turn identified more metastatic SLN in aortic area (7.9% vs. 2.4%) (p=0.070) (157). Researchers from Italy and Turkey suggest the addition of preoperative PET-CT in favor of PALAD decision (111, 113, 158). Taskin included 38 high-risk patients. Though SLN algorithm had a 100% sensitivity and NPV in finding the pelvic metastases, the IPL metastases were only detected by PET-CT. Risk factors associated with PAL metastases are reported to be type II EC, pelvic lymph node metastases, deep myometrial invasion (≥1/2) and LVSI, thus, PALAD based on these risk factors may be reasonable choice. It is noted that the detection of metastatic PAL was similar between SLN group and LAD group even in high-risk histology type EC (106, 117), which indicates that SLNM does not compromise the detection of PAL metastases in high-risk patients.

Moreover, the survival benefit of PALAD remains controversial. SEPAL study indicated that PALAD failed to affect the prognosis in low-risk patients, despite a positive impact on intermediate and high-risk patients (12); however, CART analysis conducted by Barlin et al. (159) stated that PALAD bears no relation to OS in EC patients. Whether the oncologic outcome is influenced by removing metastases directly or by personalized adjuvant therapy like radiotherapy extent based on lymph node status is unclear. Some believe that PAL metastases may be eradicated by adjuvant therapy dependent on accurate staging, which has shown to be an advantage of SLNM, which was found to detect more stage IIIC patients despite fewer lymph nodes dissected than extensive LAD.

### Non-SLN Metastasis

A major challenge in implementing SLNM lies in the potential of residual metastasis of non-SLN. Retrospective data have reported an incidence of 35% to 40% of non-SLN metastasis (64, 138). The risk of non-SLN metastasis is associated with the size of SLN metastasis and uterine higher-risk factors (160). Touhami et al. (138) found out that 60.8% of non-SLNs were positive when SLN was found to harbor macro-metastases. Otherwise, only 5% non-SLN was positive when SLN had LVMD. Similar results were reached by Biocchi, 54.5% macrometastasis and 15.4% micrometastasis were found non-SLN involvement, whereas in patients with ITCs in SLN, no metastasis was found in non-SLN (161). Turkish Gynecologic Oncology Group showed that one third SLN positive had non-SLN metastases, and the ratio increases to two thirds when SLN involvement was macrometastasis (162). Although non-SLN metastases could be controlled by adjuvant therapy and the promising results of high-risk EC patients support the hypothesis, the appropriate management of non-SLN is still worthy of further studies. Therefore, it is essential to strictly follow SLN algorithm, carefully evaluate non-SLN, and remove all suspicious enlarged lymph nodes. Further studies should be carried on to evaluate the effect of leaving metastatic non-SLNs in-situ.

## Future Directions

In summary, quantities of studies indicated that SLNM may be a safe and effective alternative for lymph node assessment in apparently uterine-confined EC with a sufficient diagnostic accuracy and similar survival prognosis even in unfavorable histology types, thus it is gaining widespread acceptance to perform SLNM in EC patients. However, the lack of convinced evidence like RCTs and long-term follow-up data limit its utilization. Further investigations should be focused on the oncologic outcomes of SLNM and the clinical relevance of LVMD on adjuvant therapy. Better standardization of SLNM protocol, surgical training program, and ultra-staging technique are also needed. Besides, further improvement in the diagnostic accuracy and therapeutic safety of SLNM are in urgent need to provide more personal and minimal-invasive treatment for EC patients and make a difference to their prognosis.

## Author Contributions

LZ drafted the manuscript. LZ was responsible for the planning and carrying out the study. LZ and XZ reviewed the literature and summarized the data. MC carefully revised the manuscript. JW was responsible for the conceptualization and final review of this manuscript. All authors contributed to the article and approved the submitted version.

## Funding

This study was supported by the National Natural Science Foundation of China (grants. 81874108, 81802607, and 82072861), the Beijing Municipal Natural Science Foundation (grant 7202213), and National Key Technology R&D Program of China (grants 2019YFC1005200 and 2019YFC1005204).

## Conflict of Interest

The authors declare that the research was conducted in the absence of any commercial or financial relationships that could be construed as a potential conflict of interest.
